# A universal mechanism of extreme events and critical phenomena

**DOI:** 10.1038/srep21612

**Published:** 2016-02-16

**Authors:** J. H. Wu, Q. Jia

**Affiliations:** 1 Peter Grünberg Research Center, Nanjing University of Posts and Telecommunications of China, Nanjing 210003, China; 2 Department of Management, Hohai University, Nanjing 211100, China; 3 Pioneer Research Center for Biomedical Nanocrystals, Korea University, Seoul 136-713, South Korea

## Abstract

The occurrence of extreme events and critical phenomena is of importance because they can have inquisitive scientific impact and profound socio-economic consequences. Here we show a universal mechanism describing extreme events along with critical phenomena and derive a general expression of the probability distribution without concerning the physical details of individual events or critical properties. The general probability distribution unifies most important distributions in the field and demonstrates improved performance. The shape and symmetry of the general distribution is determined by the parameters of the fluctuations. Our work sheds judicious insights into the dynamical processes of complex systems with practical significance and provides a general approach of studying extreme and critical episodes in a combined and multidisciplinary scheme.

Extreme events and critical phenomena commonly take place in nature and society from extraordinary occurrences to critical properties, immensely diverse in types and dissimilar in properties^1^,^2^,^3^. They can be natural and/or anthropogenic in origin and show multifaceted features of rareness, complexity and extremality^1^,^2^,^3^,^4^,^5^,^6^. The occurrence often proves awesomely challenging in forecasting and severe impact on the physical world involving loss of properties or even life, but opportunities after shake-up^1^,^2^,^3^,^5^,^6^,^7^,^8^. Such events and phenomena are pervading in a wide range of fields as global variables or quantities. Most prominent practical situations are found in self-organized critical phenomena^7^, Darwinian evolution of fitter proteins^9^, complex spontaneous brain activity^10^, fickle stock exchange^11^, conductance flux in bacteriorhodopsin films^12^, acute scenarios in capricious weather^13^,^14^, power fluctuations in electroconvection^15^, worldwide seismicity^16^ and geophysical processes^17^. Furthermore, intimately related studies and findings are dynamics in glassy systems^18^ and intermittent imbibition fronting^19^, aging research on maximum lifespan^20^, ventricular fibrillation in very short electrocardiogram episodes^21^, non-trivial criticality scaling in galaxy distribution^22^, roughing 1/f noise in a resistor^23^, instable resistance near electrical breakdown^24^, and non-equilibrium critical point in GaAs^25^, among many others.

Hence, the importance of comprehensive understanding of underlying mechanisms for the happening of extreme events and critical phenomena as well as their connection cannot be over-emphasized. As systems may be complicated, great efforts are categorically required to cope with them^1^,^2^,^3^,^4^,^5^,^6^. In fact, the fluctuating behavior in the global quantities as addressed above, though tremendously diverse in variety and different in properties, may in principle be described by the statistics of extreme values and/or sums of random variables, regardless of being independent or correlated^1^,^2^,^3^,^4^,^5^,^6^,^7^,^8^,^17^. To deal with extreme events, the first theoretical treatment is done by the Gumbel distribution (GD) on the extremal sequencing of independent and identical random variables^4^,^7^. The Gumbel distribution reads^4^,^6^


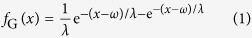


as one of the Fisher-Tippet-Gumbel (FTG) distributions (λ is a scale parameter and ω is the mode). A straightforward calculation shows that equation (1) has the mean value of *ω* + *γλ* (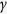 is the Euler–Mascheroni constant) and the standard deviation of 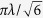. In contrast, critical behavior in purported correlated systems is similarly observed and the corresponding asymmetric distributions emerge in the way that such non-Gaussian distributions in those very different systems from turbulence to self-organized criticality, when properly rescaled, can collapse to the Bramwell-Holdsworth-Pinton (BHP) distribution^7^,


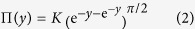


in which *y* = *b* (*x* − *s*) with *K*, *b* and *s* being parameters and π the ratio of circle’s circumference to diameter, or the generalized Gumbel (GG) distribution^8^


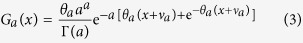


where *a* is a real positive number, 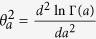, and 
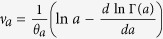
 (Γ(*a*) is the Gamma function).

By and large, the derivation of the distributions as just addressed has arisen either from extremization or concrete models, suggestive of a possible connection between a global quantity and extreme value problems associated with extreme value distributions^4^,^5^,^6^,^7^,^8^. But a clear, universal delineation which bonds the dynamical processes of different complex systems in the same vein is still elusive and under active exploitation, to satisfactorily tackle questions such as correlation between universal fluctuations of a global variable and extremal statistics, or more specifically, finding a general mapping between minmax values for extreme events and the sums of broadly distributed variables for critical phenomena^6^,^7^,^8^. Here we show a universal mechanism of extreme events and critical phenomena, disregard of a concrete system or property, to establish a general platform to bring together different systems in the same perspective. We unveil a more extended expression for the probability distribution, in which the GD, BHP, or GG distributions come out as particular cases. The properties like shape and symmetry of the general distribution are exclusively determined by the parameters of the fluctuating variables, highlighting that asymmetric fluctuations overwhelmingly matter and extreme events as well as critical phenomena can ensue. Our work shall put forward a useful framework in the research of different extreme events and critical phenomena in the extensive spectrum in the connection with ordinary events.

## Results

We first consider a global variable (quantity) of a system as the sum over infinite many random variables (fluctuations) around its mean value at different levels of stochastic cascading with exponential distributions^8^,^23^,^26^. Suppose *X*_n_ (n = 1, 2, 3, …, m, m → +∞) are these independent distributed random variables (constructing a harmonic-like series of *α*, *α* + *β*, *α* + 2*β*, *α* + 3*β*, …), with the exponential distribution of


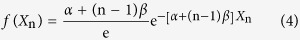


defined over the domain of 
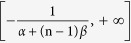
 and otherwise zero, with two parameters of *α* > 0 and *β* > 0. Evidently, *X*_n_ has the expectation of 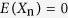 and a standard deviation (*X*_n_) or *σ*_n_ for brevity of 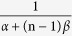. With the zero mean value and the limited magnitude of the standard deviation, *X*_n_ represents a fluctuating contribution to the global quantity of the system. The fluctuation strength of *X*_n_ may be quantified through its standard deviation. As n increases, the measure of *σ*_n_ shows a harmonic-like dwindle.

We are interested in the limiting probability density distribution of the global variable *X* defined as


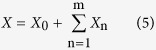


as m → +∞. In the equation, *X*_0_ is a constant random variable and has the same value as the mean (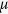) of the global variable because of *E* (*X*) = *E* (*X*_0_), or the mean value is originated from the constant random variable. Moreover, *X* has a finite standard deviation square of 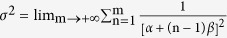 or 
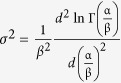
. It is a useful remark that the sum 
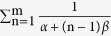
 is divergent when m → +∞, so *X* goes over the domain (−∞, +∞). For *X*_n_ is defined over a domain depending on n, it facilitates to carry out a variable transform by 
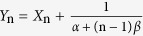
 (n = 1, 2, 3, …, m, m → +∞) as new random variables, with the modified exponential distribution of


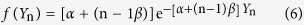


over the domain of [0, +∞] and zero elsewhere. In this case, all variables {*Y*_n_} are defined on the same domain. Obviously, *Y*_n_ has a finite mean of 
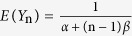
, rather than zero for *X*_n_, but the standard deviation remains the same for both *X*_n_ and *Y*_n_. Therefore, the computation of the probability of equation(5)


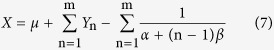


is converted to the probability estimation of 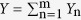 in the limit of m → +∞,


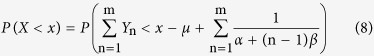


As the probability distribution of sum of many independent random variables is the convolution of each of their distributions, the probability distribution of the convolution 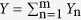, under the condition of equation (6), may be deduced to take the form of 27


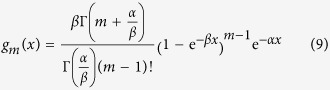


Consequently, the probability of 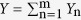 is expressible to be 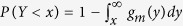 or


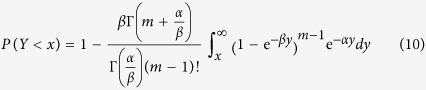


after the substitution of equation (9). To calculate the integral part of equation (10), we introduce the variable *z* = e^−*βy*^ and then obtain


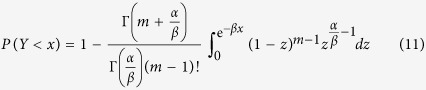


Substituting equation (11) into equation (8), we get


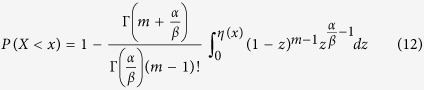


where 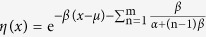. Several asymptotic expressions for the gamma and digamma functions are required to compute equation (12) 28, that is, 
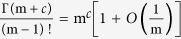
, 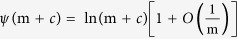 (here 
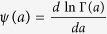
 being the digamma function) and 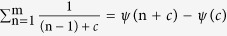. Thus, 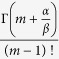 is simplified to 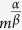, and 
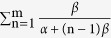
 in *η*(*x*) turns out to be 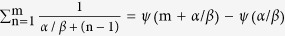 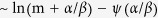. As a result, 
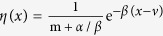
, with 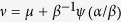.

After the asymptotic changes, we may formulate equation (12) in an asymptotical expression as


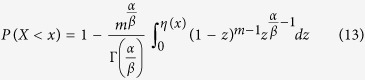


To simplify the manipulation and get the limiting formulas, we make a new variable substitution by *τ* = *mz* and use the relation of 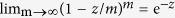. Subsequently, equation (13) is transformed to


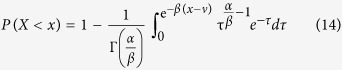


By directly differentiating equation (14), we in effect arrive at the general probability distribution function of the global quantity *X* as


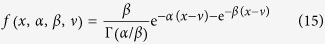


or equivalently,


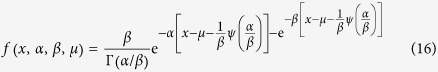


with the mode at 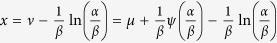. A straightforward check shows that *f* (*x*) meets the conditions as a probability density function with three parameters.

## Discussion

In terms of equation (15), the major probabilistic properties of the global variable are solely determined by the properties of the infinite many small asymmetric fluctuations around the mean value from the constant random variable, characterized by the parameters, 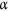 and 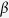, describing the fluctuations. The mean, 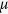, shifts the location of the probability density distribution, but does not affect the shape and symmetry of the distribution. Moreover, 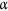 and 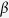 may contribute to the location of the distribution by a magnitude of 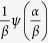.

We come to discuss several special cases of this more extended probability distribution of equation (15). As indicated that the Gumbel distribution of equation (1) is first proposed for the successful description of extreme events^1^,^4^,^8^, we show that equation (1) can be obtained from equation (15). In point of fact, if we set *α* = *β* = 1/*λ* and *ν* = *ω*, equation (15) becomes equation (1), in other words, we may get the Gumbel distribution from the new general distribution^4^,^8^.

A broad variety of critical phenomena have been described quite satisfactorily by the BHP distribution of equation (2) or the GG distribution of equation (3). For example, the distribution of equation (2) or equation (3) is capable of describing the fluctuations of a global quantity in correlated equilibrium and nonequilibrium systems, from 2D XY model, modeling of forest fires, the dynamics of sandpiles and avalanches, percolation phenomena, Ising and a coupled rotor models, and self-organized critical granular media. Nevertheless, we have pointed out that these valuable, well-founded distributions may be derived from the general probability distribution of equation (15) or equation (16) as proposed in this work. If we put *α*/*β* = π/2, *β* = *b*, 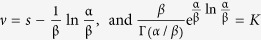, we may recover the BHP distribution equation (2) from equation (15) 7,8. If we set *α*/*β* = *a*, *β* = *θ*_*a*_, and 
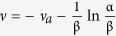
, we may obtain the GG distribution expressed in equation (3) from equation (15) 8. Accordingly, the extended distribution of equation (15) is a more general probability distribution, including the GD, BHP, or GG distributions as special cases, which can describe extreme and critical events from exceptional episodes to criticality concerned.

**Figure 1:**
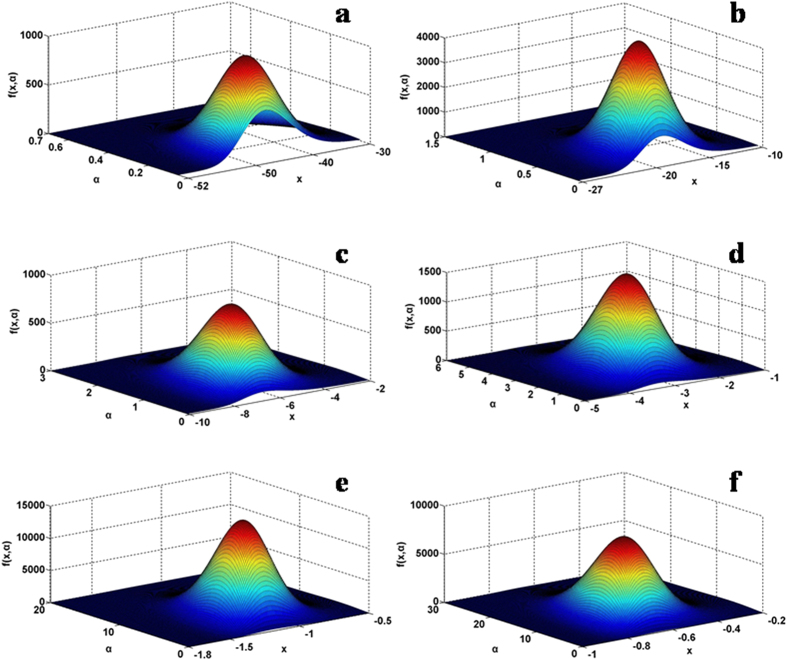
3D Plots of the general probability density distribution as a function of the variable *x* as well as the parameter *α* under the conditions of *μ* = 0 and a specified *β* value. (**a**) *β* = 0.1. (**b**) *β* = 0.2. (**c**) *β* = 0.5. (**d**) *β* = 1.0. (**e**) *β* = 3.0. (**f**) *β* = 5.0.

There are three parameters in the general probability density distribution, so plotting the function *f* (*x*, *α*, *β*, *μ*) or *f* (*x*, *α*, *β*, *ν*) is of necessity to restraint the parameters. As previously noted, the change of the mean *μ* may translate the relevant curve horizontally, rather than the symmetry and shaping characteristics, we may set it to zero in the elucidation below. Consequently, we have the two remaining parameters, 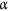 and 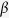, to tackle. The appreciation of the effects of the parameters on the behavior of the general probability density distribution is revealed by dividing the parametric space in particular scenarios. In Fig. 1, the 3D plots are calculated by *f* (*x*, *α*, *β*, *μ* = 0) = 
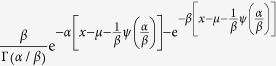
 as a function of *x* and *α* for a specified 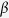 from 0.1, 0.2, 0.5, 1.0, 3.0 to 5.0 (more examples are given in the Supplementary Information). Overall, the probability distribution shows cap-like surface mapping and the sliced section along *x*-axis at a given *α*, namely, a probability distribution curve as a function of *x* at the chosen *α* and *β*, monotonically increases, reaches the maximum and then decreases. The skew shape and symmetry is clearly dependent on the *α* parameter and the location of the sliced curve shifts with *α*. In addition, the position of the peak value (the mode) is evidently dependent on *α* and *β* in general, as determined by 
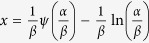
 when 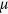 is set to 0.

**Figure 2:**
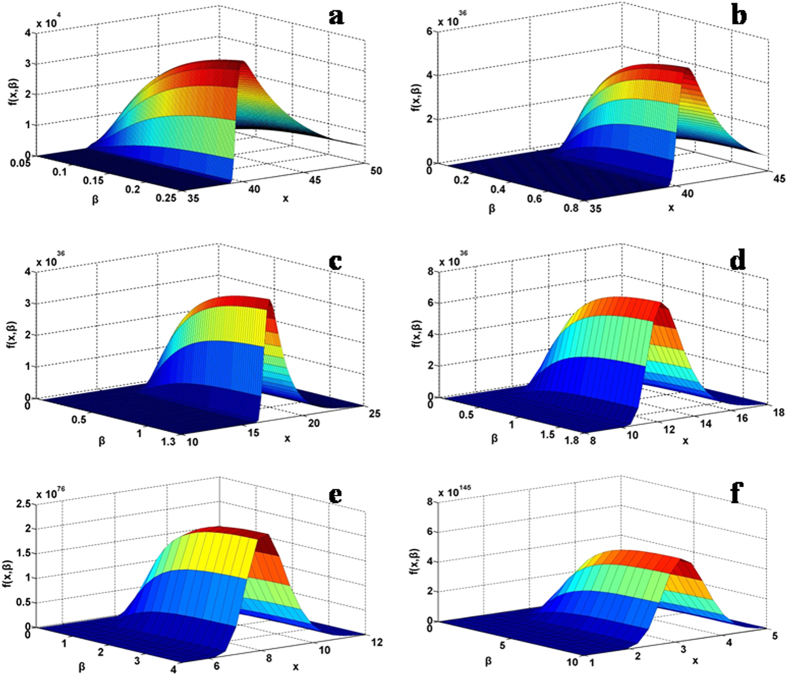
Plots of the general probability density distribution as a function of the variable *x* as well as the parameter *β* under the conditions of *μ* = 0 and a specified value of *α*. (**a**) *α* = 0.2. (**b**) *α* = 0.5. (**c**) *α* = 0.8. (**d**) *α* = 1.0. (**e**) *α* = 2.0. (**f**) *α* = 5.0.

In the same vein, 3D plots are obtained by considering 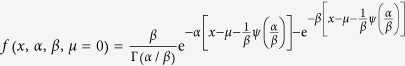 as a function of *x* and *β* for a specified *α* from 0.2, 0.5, 0.8, 1.0, 2.0 to 5.0, as shown in Fig. 2 (more examples are given in the Supplementary Information). The probability distribution demonstrates camping-tent-like or half snail-like surface diagrams. Analogous to Fig. 1, a sliced section along *x*-axis for a selected 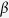, i.e., a probability distribution curve at the given *α* and *β*, monotonically increases, attains the peak value and then declines. Obviously, the parameters *α* and *β* have different effects on the behavior of the general probability distribution.

**Figure 3:**
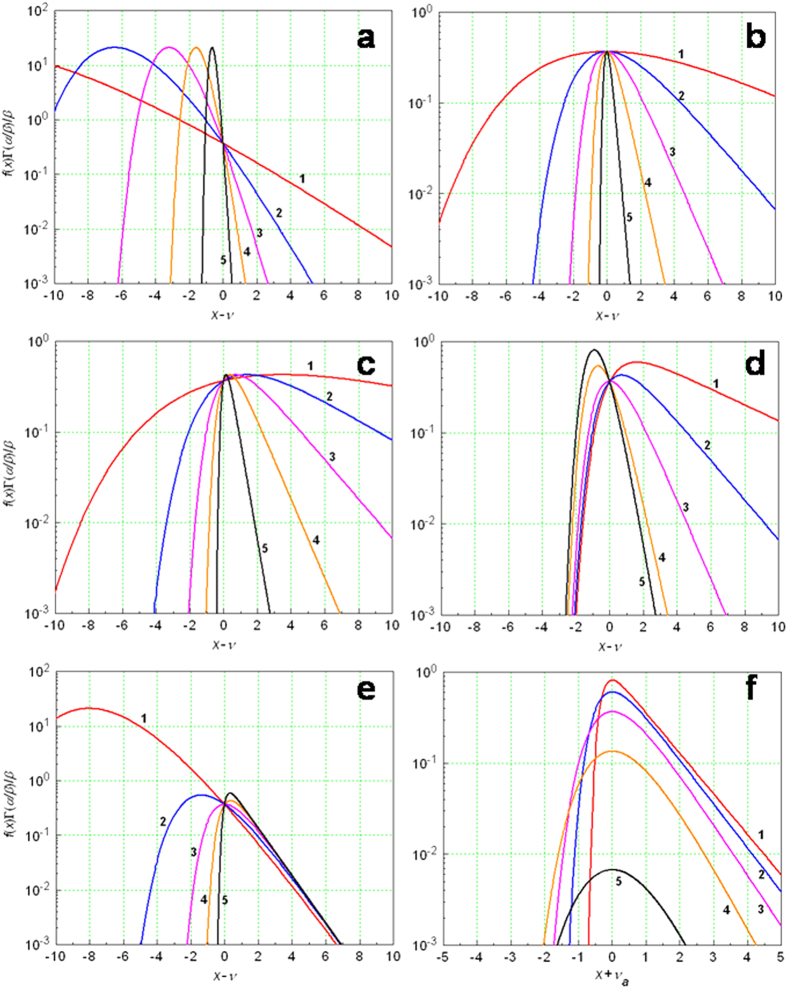
Effects of the parameters *α* and *β* on the behavior of the general probability density distribution. (**a**) Curves 1 ~ 5 correspond to *α* ∈ (0.5, 1.25, 2.5, 5, 12.5) for *α*/*β* = 5. (**b**) Curves 1 ~ 5 indicate the plots of *α* ∈ (0.2, 0.5, 1, 2, 5) for *α*/*β* = 1. (**c**) Curves 1 ~ 5 illustrate *α*/*β* = 1/2 and *α* ∈ (0.1, 0.25, 0.5, 1, 2.5). (**d**) Curves 1 ~ 5 are the results of setting *b* = 1 and *α* ∈ (0.2, 0.5, 1, 2, 2.5). (**e**) Curves 1 ~ 5 describe the outcomes of setting *α* = 1 and *β* ∈ (0.2, 0.5, 1, 2, 5). (**f**) Curves 1 ~ 5 show the GG distribution for *α* ∈ (0.2, 0.5, 1, 2, 5).

A useful, more direct intuition of the behavior of the universal probability density distribution is attained in 2D plotting by further fixing *α* or *β*, as shown in Fig. 3. In the calculation of the curves, the general probability density distribution function equation (15) is used and the curves are presented in the scaled form of *f* (*x*, *α*, *β*, *ν*)Γ(*α*/*β*) vs. (*x* − *ν*). Figure 3a shows the case of *α*/*β* = 5 (*α* > *β*) and *α* ∈ (0.5, 1.25, 2.5, 5, 12.5). The function strongly depends on the parameters and tightens the distribution as the parametric values increase. More symmetric distribution is found with the case of *α*/*β* = 1 (*α* = *β*) and *α* ∈ (0.2, 0.5, 1, 2, 5), as given in Fig. 3b. Figure 3c illustrates the situation of *α*/*β* = 1/2 (*α* < *β*) and *α* ∈ (0.1, 0.25, 0.5, 1, 2.5), revealing a similar narrowing spread of the distribution with an increasing value of *α*. We then fix one of the two parameters, 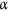 or 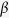. Figure 3d is the result of setting *β* = 1 and *α* ∈ (0.2, 0.5, 1, 2, 2.5), characterized by the collapse of the curves on the narrow zone on the left. In contrast, Fig. 3e shows the outcome of setting *α* = 1 and *β* ∈ (0.2, 0.5, 1, 2, 5), featured by the close descendency of the curves on the narrow zone on the right. The GG distribution is comparatively displayed in Fig. 3f, with the parametric selection of *α* ∈ (0.2, 0.5, 1, 2, 5), which is in conformity with the characteristics of the curves from the new general distribution. More examples are shown in the Supplementary Information.

**Figure 4:**
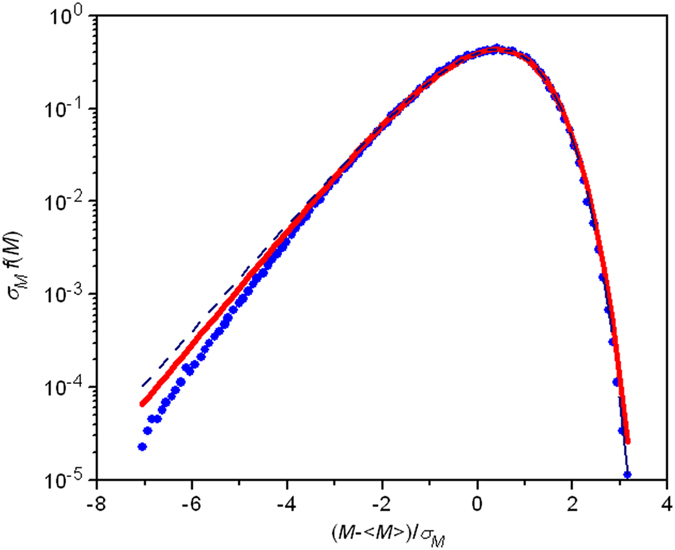
Comparative analyses of the general probability density distribution and experimental data. Solid circles: Experimental data. Real line: Fit by equation (15). Dash line: Fit by equation (2).

As addressed above, extreme events alongside critical phenomena may have been appropriately elucidated by the GD distribution of equation (1) and the BHP distribution of equation (2) or the GG distribution of equation (3). As a result, improved effect is expected from the newly derived universal distribution. For substantiation, the experimental data of 2D XY modeling were comparatively evaluated by equation (15) vs. equation (2) 29. As shown in Fig. 4, the function of equation (15) (real line) adequately describes the experimental data (solid circles), much better than that of equation (2) (dash line). The parameters as obtained from the calculation of equation (15) are *α* = 1.42078, *β* = 0.78065 and *ν* = 1.42078 when the magnetization is interpreted as a negative variable instead. The derived exponent from equation (15) is 1.8200, much larger than π/2^29^. We point out that both the GG distribution of equation (3) and the BHP distribution of equation (2) have an analogous form, but the former seems to have less freedom than the latter. So, it is expected that the GG distribution may have a poorer fit of the data.

Since the extended distribution of equation (15) as a more general probability distribution function is derived from the two-parametric fluctuations as well as the mean value of the global quantity, it is of interest to elaborate a bit more about the choice of the parameters and the consequent reduction to a specific probability density function. As mentioned above, the peak value of equation (15) is specified at 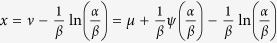, so all the three parameters together determine the maximum density position of the distribution, showing the non-Gaussian characteristics of the global quantity. As shown in Figs 1, 2, 3, the skew shape and asymmetric attribute of the probability density distribution in contrast is obviously dependent on the selection of the *α* and *β* parameters but not the mean, 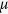. When we choose the parameters under the condition of *α* = *β*, the fluctuations are reduced to one-parametric ones, a series of *α*, 2*α*, 3*α*, …, and correspondingly, equation (15) turns to be the GD distribution describing extreme events. Alternatively, the extreme events can be equivalently handled in the context of both extreme value theory and special correlated cases. Nonetheless, it is quite obvious that the condition of *α* = *β* isn’t satisfied in general, so that the parameters are not reducible to the same single one, giving the description of correlated events.

The approach and results as obtained in this work may have broad implications and useful applications in addressing extreme events and correlated phenomena since pertinent physical systems may be appropriately represented by global quantities (variables), for example, fluid turbulent transport, percolation phenomena, modeling of forest fires, 2D XY model, Darwinian evolution of fitter proteins, spontaneous brain activity, stock exchange, capricious weather, and seismicity. As a matter of fact, a global quantity (variable) which is handled as the statistics of sum of infinite many random variables about its mean value is a proper descriptor of additive properties of a physical system, such as magnetization and total energy, and the corresponding probability distribution function is of significance to manifest information on the physics of the system. In the case of the 2D XY model as previously discussed, spins fluctuate around their mean value as spin magnetic momentum variables *X*_n_ with the expectation of *E* (*X*_n_) = 0 according to the asymmetric exponential distributions of equation (4), in which the constants have a definite relation with respect to the two parameters of *α* and *β*, featuring the characteristic of a correlated system. The magnetization of the model system has proved to follow the general probability distribution function of equation (15) (Fig. 4), revealing a common feature of an extreme event and a correlated system. Clearly, this approach, when suitable global variables are selected, is extendable to other physical systems, including the situations which have been successfully elucidated by the Gumbel distribution (GD), the Bramwell-Holdsworth-Pinton (BHP) distribution, and the generalized Gumbel (GG) distribution. It may be worthwhile to stress that the probability distribution function of a global quantity of a physical system is uniquely governed by the characteristics of its local fluctuating variables.

It may be constructive to give a brief comparison between previous fruitful investigation (cf. ref. 6 and references cited therein) and our work. We first point out that our approach is original and more general. The corresponding result is new, based on the sound mathematical derivation and supported by experimental data. For instance, equation (15) in our work isn’t reported in the precedent publication, which is a more general formula for extreme value events and correlated phenomena. The assumption we have used is exponential fluctuations, but Gaussian or other tail fluctuations are used in the previous papers. Moreover, our derivation is mathematically justified and the expression is exact, in contrast to possible use of numerical analyses and even heuristic arguments in the previous papers and that the derivation is usually proceeded case by case or discussed in different values of α in the case of 1/f^α^ noise, with results expressed in the sum or product of terms of some infinite series which is hard to evaluate. Furthermore, we have demonstrated the fitting of equation (15) to experimental data better than other distributions, while no such comparison is done in other researches yet. A more significant difference lies in the fact that, as presented in the above, the general probability distribution function of equation (15) or equation (16) we have initiated can take well-known extreme distributions as particular cases, but not otherwise.

In conclusion, the general probability distribution of equation (15) or (16) may pertinently unify the most famous probability distributions in the subject and shows a universal mechanism of extreme events and critical phenomena. The behavior of the global variable is dictated by the superimposing dynamical interaction of the infinite many asymmetric fluctuations in the system. Our approach to deriving the general probability distribution can cast light on imminent issues like climate change, stock exchanges and earthquake on the important role of fluctuations on the devastating dynamical processes and provides a more general unifying and multidisciplinary frame underpinning the study of widespread, different extreme events and critical properties. It offers new thinking and opportunities for the study, risk management and control of extreme events and critical phenomena.

## Methods

### Plotting of the general equation

As the general equation (15) or (16) has three parameters, *α*, *β*, and *μ*, 3D plotting requires to fix two of them. Since *μ* is the mean in the probability distribution and shifts the location of the corresponding curve but not the shape feature or symmetric property, so we set it to zero. Then, we fix one of the remaining two parameters, *β* at several discrete values and plot the distribution as a function of *x* and *α*. In turn, we set *α* at a fixed value and then plot the distribution as a function of *x* and *β*. Explicitly, in the calculation of Figs 1 and 2, the following function of the general probability density distribution is used and *μ* is set to 0,


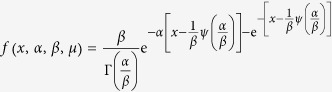


Particularly, in the calculation of Fig. 1, 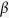 is set to a given number and then *f* (*x*, *α*, *β*, *μ*) is calculated as a function of *x* and *α*. In the calculation of Fig. 2, *α* is set to a specified number and then *f* (*x*, *α*, *β*, *μ*) is calculated as a function of *x* and 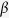.

2D plotting is given to get more intuitive comprehension of the behavior of the universal probability density distribution by further fixing *α* in Fig. 1 or *β* in Fig. 2 Overtly, in the calculation of Fig. 3, the following function of the general probability density distribution is used,


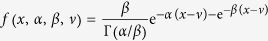


The curves were plotted with relative locations of *x*-*ν* and scaled probability densities of 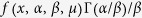. For the GG distribution, the curves are presented in the form of 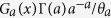 vs. *x* + *ν*_*a*_.

### Comparison analysis of experimental data

To evaluate the improved functioning of the newly derived universal distribution, the experimental data were extracted from the documented report^29^ and equation (15) vs. equation (2) was comparatively examined. The magnetization (*M*-<M>)/*σ*_*M*_ is interpreted as a negative variable *x* in equation (15) in accordance with the positive definition of the parameters, *α* and *β*. The probability density is scaled by a magnitude of *σ*_*M*_.

## Additional Information

**How to cite this article**: Wu, J. H. and Jia, Q. A universal mechanism of extreme events and critical phenomena. *Sci. Rep.* **6**, 21612; doi: 10.1038/srep21612 (2016).

## Supplementary Material

Supplementary Information
