# Corrigendum: A universal mechanism of extreme events and critical phenomena

**DOI:** 10.1038/srep23874

**Published:** 2016-04-20

**Authors:** J. H. Wu, Q. Jia

Scientific Reports 6: Article number: 2161210.1038/srep21612; published online: 02162016; updated: 04202016.

This Article contains typographical errors in the Acknowledgements section.

“Jiangsu Provincial Natural Science Foundation of China (No. 1063-515024111)”

should read:

“Jiangsu Provincial Natural Science Foundation of China (No. BK20150823)”

