# Supplementary material for: A universal mechanism of extreme events and critical phenomena
Source: Sci Rep. 2016 Feb 16;6:21612. doi: 10.1038/srep21612 (PMC4754907; doi:10.1038/srep21612)
Supplement: Supplementary Information [file srep21612-s1.docm]

**A universal mechanism of extreme events and critical phenomena**

J. H. Wu^1,2^ & Q. Jia^3^

**Supplementary Information**

In the calculation of Figs. 1 & 2, the following function of the general probability density distribution is used and is set to 0,

$$f\left( x,,, \right)=\frac{}{\Gamma\left( \frac{}{} \right)}e^{-\left[ x-\frac{1}{}\psi\left( \frac{}{} \right) \right]-e^{-\left[ x-\frac{1}{}\psi\left( \frac{}{} \right) \right]}}$$

In the calculation of Fig. 1, is set to a given number and then $f(x,,,)$ is calculated as a function of *x* and . In the calculation of Fig. 2, is set to a specified number and then $f(x,,,)$ is calculated as a function of *x* and .

In the calculation of Fig. 3, the following function of the general probability density distribution is used,

$$f(x,,,)=\frac{}{\Gamma(/)}e^{-\left( x- \right)-e^{-\left( x- \right)}}$$

In the plots, the curves are presented in the scaled form of $f(x,,,)\Gamma(/)/$ vs. $(x-)$.

**Figure S1** | 3D **Plots of the general probability density distribution as a function of the variable *x* as well as the parameter under the conditions of** $\boldsymbol{=0}$ **and a specified value.**  **a,** $=0.3$. **b,** $=0.4$. **c,** $=0.6$. **d,** $=0.9$. **e,** $=2.0$. **f,** $=4.0$.

**Figure S2** | **Plots of the general probability density distribution as a function of the variable *x* as well as the parameter under the conditions of** $\boldsymbol{=0}$ **and a specified value of .** **a,** $\alpha=0.3$. **b,** $\alpha=0.4$. **c,** $\alpha=0.6$. **d,** $\alpha=0.9$. **e,** $\alpha=3.0$. **f,** $\alpha=4.0$.

**Figure S3** | **Effects of the parameters and on the behavior of the general probability density distribution.** **a,** Curves 1~5 correspond to $\alpha$ (0.1, 0.25, 0.5, 1, 2.5) for $/=1/5$. **b,** Curves 1~5 indicate the plots of $\alpha$ (0.1, 0.8, 1.5, 3, 10) for $/=1$. **c,** Curves 1~5 illustrate $\alpha$ (0.2, 0.5, 1, 2, 5) for $/=2$. **d,** Curves 1~5 are the results of setting $=0.5$ and (0.2, 0.5, 1, 2, 5). **e,** Curves 1~5 describe the outcomes of setting $=0.5$ and $\alpha$ (0.2, 0.5, 1, 2, 2.5). **f,** Curves 1~5 show the plots under the conditions of setting $=1.5$ and $\alpha$ (0.2, 0.5, 1, 2, 2.5).
